# An Unusual Cause of Acute Heart Failure: A Case Report of Iliocaval Venous Stent Migration

**DOI:** 10.1177/2324709618799118

**Published:** 2018-09-07

**Authors:** Sherif Elmahdy, Christian C. Shults, Muhamad Alhaj Moustafa

**Affiliations:** 1 MedStar Washington Hospital Center, Washington, DC, USA

**Keywords:** stent migration, heart failure, May-Thurner syndrome, tricuspid valve replacement, common iliac vein

## Abstract

Stent migration is an uncommon complication of endovascular stenting procedures. It could cause serious cardiovascular complications. In this article, we describe an interesting case of acute heart failure as a result of stent embolization from the left common iliac vein into the right ventricle and how it was identified and managed.

## Introduction

Stent migration is dislodgement of the endoprosthesis from the primary intended deployment site and is considered an uncommon but conceivably fatal complication. The incidence of stent migration as a complication of endovascular stenting has been reported to be as low as 0.9% of all percutaneous stenting procedures.^1^ The majority of cases reported with stent embolization to the right heart were from the central veins of the thorax, upper extremity veins from dialysis fistulas, or renal veins for nutcracker syndrome.^2-4^ Stent migration to the right heart from iliac veins is seldom reported.^5-7^ Endovascular extraction is the preferred initial intervention. However, some cases require surgical retrieval, especially if there is valvulopathy requiring correction or involvement of the subvalvular apparatus. Here, we discuss a case of acute-onset heart failure as an initial presentation of stent migration and how it was identified and treated.

## Case Description

A 61-year-old female with medical history of hypertension, diabetes mellitus type 2, and chronic kidney disease stage V was transferred to our institution from an outside hospital for further evaluation and definitive management of a migrated intracardiac stent.

**Figure 1.**
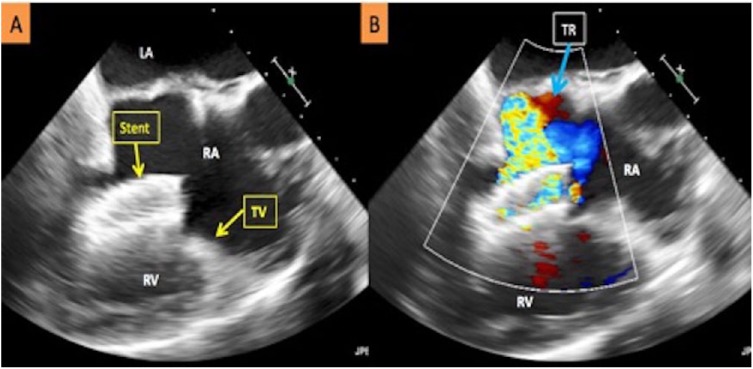
(A) Transesophageal echocardiogram depicting the migrated stent across the tricuspid valve resulting in severe tricuspid regurgitation. (B) Doppler-enhanced transesophageal echocardiogram image depicting severe tricuspid regurgitation as a result of the migrated stent. LA, left atrium; RA, right atrium; RV, right ventricle; TV, tricuspid valve; TR, tricuspid regurgitation.

She initially presented to the outside facility with progressive dyspnea on exertion, orthopnea, and bilateral lower extremity edema. She was initially diagnosed with acute heart failure and pneumonia and treated with diuretics and antibiotics. Subsequently, a transthoracic echocardiogram was performed, which revealed a foreign body within the right ventricle. On transfer to our facility, a transesophageal echocardiogram revealed a long stent straddling the tricuspid valve from the right atrium with the other end lodged in the trabeculation of the right ventricle with severe tricuspid regurgitation (Figure 1).

On further investigation, we learned that the patient had undergone peripheral endovascular intervention for May-Thurner syndrome with placement of a self-expanding Nitinol Protege (14 mm × 60 mm) stent to the left iliac vein 6 months prior to presentation.

A percutaneous endovascular approach with a 35- mm Medtronic-Covidien Amplatzer Gooseneck Snare was initially attempted to retrieve the migrated stent. However, the snared proximal segment fractured, leaving behind 2 stent fragments. After ensuring there was no myocardial perforation or pericardial effusion with intracardiac ultrasound, the patient was referred for surgical extraction via median sternotomy with use of cardiopulmonary bypass.

**Figure 2.**
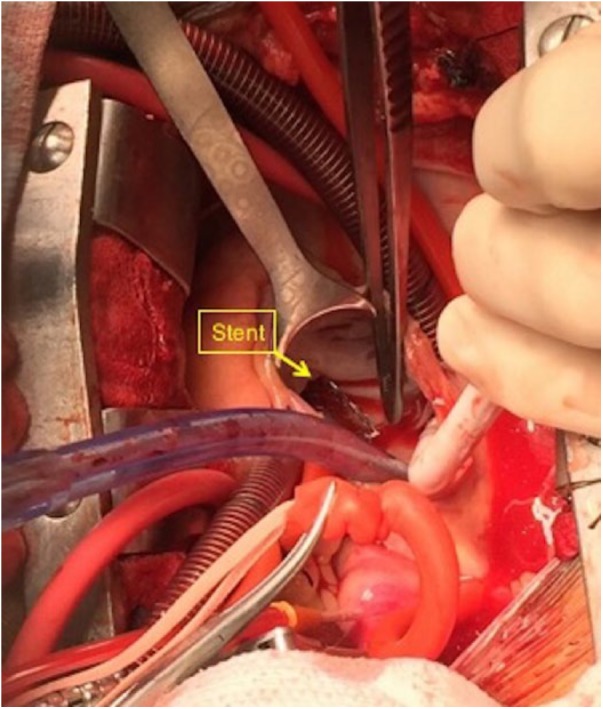
Intraoperative image showing surgical retrieval of the embolized stent through open sternotomy assisted by cardiac bypass.

During the operative procedure, the stent was found to be densely adherent to the tricuspid leaflets and the subvalvular apparatus, with majority of the primary chords to the anterior and posterior leaflets ruptured (Figure 2). After successful extraction of the stent and native tricuspid valve, she underwent valve replacement with a 29-mm Carpentier-Edwards bioprosthetic valve. Her postoperative course was complicated by hemopericardium secondary to anticoagulation resulting in cardiac tamponade that was drained percutaneously, and small thromboembolic cerebellar stroke from atrial fibrillation. She was discharged to an inpatient rehabilitation facility and did well on 8-month follow-up.

## Discussion

Stent migration is a rare cause of acute heart failure and valvulopathy. However, it should be suspected promptly in patients with history of venous stent placement. Early diagnosis and treatment may prevent major complications and death. Stent migration may cause vessel rupture, cardiac conduction abnormalities, valvular destruction, and/or myocardial perforation. Our patient had May-Thurner syndrome, which is an acquired stenosis of the left common iliac vein resulting from compression of the vein against the lumbar vertebrae by the overlying right common iliac artery. This phenomenon was first described by Robert May and Josef Thurner in Austria in 1957. They examined 430 cadavers and reported that 22% of the cases had a spur-like structure in the common iliac vein caused by chronic compression on the vessel. These spur-like formations are deemed to be the cause of the thrombosis as they disturb the blood flow. The authors also found that the thrombosis was 8 times more common on the left side due to anatomic differences.^8^ First-line therapy for this syndrome is balloon dilation and stenting, which have been found to be superior to conventional surgical treatment,^9^ with 1-year stent patency rates approaching 100%.^10,11^ Although venous stenting is seemingly similar procedure to arterial stenting, it is done less frequently and needs more experience. Venous system has different properties compared with the arterial system, which make stents less stable and prone to relocation. There are many postulated causes for venous stent migration. It can be due to stent undersizing, poor stent-venous wall contact, increased venous vascular compliance in comparison with the arterial system, the natural venous flow from smaller to larger diameter vessels, stent thrombosis that leads to flow impairment, trauma to the site, massage over the underlying stent, venous intraluminal irregularities, prestenotic iliac vein dilation that results in drifting of the stent forward, and the arterial pulsatility and pressure over the vein that could cause stent dislodgement over time. Selection of the correct stent size is a key determinant of procedural success, and the diameter of the stent must be greater than that of the designated vein to prevent stent migration and the need for unnecessary subsequent procedures. Earlier realization of acute heart failure symptoms in patients with recently placed venous stents can possibly lead to more timely detection of stent embolization to the heart and prevent fatal complications.

In summary, even though stent migration is a rare occurrence, embolization to the right atrium, ventricle, and pulmonary circulation can be catastrophic. Thus, we suggest more research should be focusing on standardizing sizing and placement techniques. We also advocate for serial imaging to be considered to confirm adequate long-term placement in order to avoid life-threatening outcomes.
